# Functionally distinct tendon fascicles exhibit different creep and stress relaxation behaviour

**DOI:** 10.1177/0954411913509977

**Published:** 2013-11-27

**Authors:** Jennifer H Shepherd, Kirsten Legerlotz, Taylan Demirci, Christian Klemt, Graham P Riley, Hazel RC Screen

**Affiliations:** 1 Institute of Bioengineering, School of Engineering and Materials Science, Queen Mary, University of London, London, UK; 2 School of Biological Sciences, University of East Anglia, Norwich, UK

**Keywords:** Tendon, mechanics, fatigue, human semitendinosus, bovine

## Abstract

Most overuse tendinopathies are thought to be associated with repeated microstrain below the failure threshold, analogous to the fatigue failure that affects materials placed under repetitive loading. Investigating the progression of fatigue damage within tendons is therefore of critical importance. There are obvious challenges associated with the sourcing of human tendon samples for *in vitro* analysis so animal models are regularly adopted. However, data indicates that fatigue life varies significantly between tendons of different species and with different stresses in life. Positional tendons such as rat tail tendon or the bovine digital extensor are commonly applied in *in vitro* studies of tendon overuse, but there is no evidence to suggest their behaviour is indicative of the types of human tendon particularly prone to overuse injuries. In this study, the fatigue response of the largely positional digital extensor and the more energy storing deep digital flexor tendon of the bovine hoof were compared to the semitendinosus tendon of the human hamstring. Fascicles from each tendon type were subjected to either stress or strain controlled fatigue loading (cyclic creep or cyclic stress relaxation respectively). Gross fascicle mechanics were monitored after cyclic stress relaxation and the mean number of cycles to failure investigated with creep loading. Bovine extensor fascicles demonstrated the poorest fatigue response, while the energy storing human semitendinosus was the most fatigue resistant. Despite the superior fatigue response of the energy storing tendons, confocal imaging suggested a similar degree of damage in all three tendon types; it appears the more energy storing tendons are better able to withstand damage without detriment to mechanics.

## Introduction

Many painful tendon lesions are thought to be the result of a gradual accumulation of micro-injuries.^1^ Tendinopathy is thus often referred to as an overuse pathology and can, in some ways, be considered analogous to the fatigue failure of common engineering materials during cyclic loading.^2^ This is of course an oversimplification, as tendon is not simply a fibre-reinforced composite, but a living tissue maintained by its resident cell population, the tenocytes. Damage to tendon probably occurs every day as a result of a variety of normal activities, but this damage only accumulates when equilibrium cannot be maintained between the rate of damage and the cellular driven rate of repair.^3^ The progression of this damage can be challenging to monitor in situ, and as such, in vitro fatigue testing routes are of significant importance for understanding overuse damage in tendons.

Tendon mechanics are defined by the hierarchically organised collagen within the tissue, providing a uniaxially strong material, in which failure of one or a few collagen components within the hierarchy does not significantly reduce the strength of the whole tendon.^4^ The fascicular level is particularly critical for the mechanics of tendon and is at a scale that can be mechanically tested with relative ease. The ability of the fascicles to act as apparently independent structural units, and slide against each other, enables them to transmit tension despite the changing angles of a joint and allows tendons to change shape as their muscle contracts.^5^ However, sliding is not just limited to between collagen units at the fascicle level; fibre straining and inter-fibre matrix shearing within the fascicle are suggested to be critical for tendon function.^6^ While fascicle mechanics cannot necessarily be considered to be representative of tendon behaviour as a whole, it can add much to the understanding of tendon mechanics and the role of the hierarchical structure.

While all tendons play a positional role, positioning joints to facilitate movement, there are also those which must operate as energy stores to allow for more efficient locomotion.^7^ Energy-storing tendons experience high strains and consequently high stresses as they stretch during loading, ready to recoil and return energy to the system. These tendons tend to operate with low safety factors and thus have a much higher propensity for injury.^8^ Prime examples of these energy-storing tendons are the human Achilles or equine superficial digital flexor tendon (SDFT), but evidence would suggest that the tendons of the musculotendon hamstring system also exhibit energy-storing capability^9,10^ with a stretch-shortening cycle observed.^11,12^ Certainly, there is a high propensity for hamstring injury and in a study that considered 170 consecutive cases of hamstring strain in athletes, 21 exhibited proximal tendon injury, 4 of these being distal tendon avulsions and 3 of which were of the semitendinosus.^13^ While not on the same scale as the equine SDFT, the peak strain of the semitendinosus musculotendon unit has been observed to be 8.73% and this musculotendon unit also observed the greatest lengthening velocity of three units of the hamstring.^11^ While positional tendons such as rat tail tendon or the bovine digital extensor are commonly applied in in vitro studies of tendon overuse, there is no evidence to suggest that their behaviour is indicative of the types of human tendon particularly prone to overuse injuries. It is likely that more energy-storing tendons from other animal models such as the equine SDFT or bovine digital flexor may prove more comparable.

In vivo loading conditions of tendon are complex, and thus, careful consideration of the nature of the fatigue loading is required, to provide the greatest equivalence to the clinical situation. Fatigue testing can be carried out either in the form of repeated loading or in the form of repeated deformation, that is, under load or displacement control.^14^ In the nomenclature of static testing, a creep test is one where a constant load is applied and the increase in extension monitored, while stress relaxation describes a test at constant displacement where the reduction in associated load is measured. However, the terms more accurately describe the materials response to the test, that is, a lengthening with constant load in the case of creep or a reduction in stress with constant displacement in the case of stress relaxation. As these responses also occur during cyclic loading,^15,16^ there has become some precedent to describe cyclic tests under either load or displacement control as cyclic creep or stress relaxation tests, respectively,^16,17^ and it is these terms that will be used here. With different boundary conditions, the two testing routes would be expected to elicit a different response from the loaded tendon, but they are often used interchangeably with very little literature comparing the two testing techniques directly. Stress relaxation tests of tendon fascicles are typically more straightforward to carry out, but as creep tests may be more physiologically relevant, they cannot simply be neglected. It has been suggested in ligament (where the function is to connect bone to bone) that the in-life loading is a simple creep situation, with the same loads repeatedly applied by the joint. However, tendons transfer the muscle force to the bone in order to facilitate locomotion, and as a result, load conditions may be more complex.^18^ The time-dependent properties of tendon will undoubtedly affect its ability to convert muscle contraction into skeletal movement, and as such, both creep and stress relaxation behaviour must be well defined in order to accurately predict the behaviour of tendon.^19^

Comparative study on the fatigue behaviour of contrasting tendon types is limited;^20,21^ yet, the behaviour of other mammalian tendon of a variety of types is frequently used to understand the progression of fatigue damage in human tendon. It is critical to have an understanding of how fatigue behaviour may differ in functionally distinct tendons and also to establish whether we are indeed increasing our understanding of human tendon behaviour by investigating other tendon types whether in vivo or in vitro. While it might be expected that the structure and mechanics of different tendons differ so as to mirror the quantitative functional variations, the evidence in the literature does not entirely support this. When Pollock and Shadwick^22^ measured Young’s modulus (E) and percentage hysteresis loss (H) for mature tendons from 18 species of mammal, individual tendons were found to differ significantly, but there was no significant relationship with the type of tendon or mass of the animal. As summarised in a review article by Ker et al.,^20^ numerous studies have also shown a lack of correlation between the ultimate tensile strength (UTS) of tendons and the stresses seen in vivo. High stress-in-life tendons such as the Achilles tendon actually tend to exhibit lower failure strengths than more positional tendons (a compromise to allow higher elasticity),^20,23–26^ meaning a lower safety margin and perhaps the higher propensity for damage. However, when fatigue properties are considered, high stress-in-life tendons show significantly greater lifetimes under cyclic loading when all tendons are fatigued to the same stress.^20^

This article investigates the fatigue characteristics of the fascicles from three tendons for the first time: the extensor and deep flexor digital tendons of the bovine hoof and the human semitendinosus tendon. The bovine digital tendons are readily available and provide a straightforward source of tendon material for in vitro investigation. Both the deep digital flexor tendon and human semitendinosus provide some energy-storing capability while the bovine digital extensor tendon plays a purely positional role. Considering fascicles from all three tendons allows the comparison of not only energy-storing and positional tendons from a single species, but also the comparison of energy-storing tendons across species, comparing the bovine deep digital flexor tendon with the clinically relevant human semitendinosus. It is hypothesised that species variation may be less significant than functional requirement in determining fascicle fatigue behaviour, and that positional tendons such as the bovine digital extensor will not provide an ideal model for investigating energy-storing and injury-prone tendons such as the hamstring semitendinosus or Achilles. Finally, the complex loading environment of tendon means that value can be gained by comparing the response to cyclic creep and cyclic stress relaxation in an in vitro study. The microstructural processes that take place during the two testing routes are likely be fundamentally different, and as such, a difference in the rate of damage progression is expected between the two fatigue conditions.

## Materials and method

### Tendon preparation

The feet of young healthy bovines (male steers between 18 and 36 months of age), with no observed tendon injury, were sourced from a local abattoir. The tensional regions of the medial, lateral and common digital extensor tendons (CDETs) as well as the deep digital flexor tendon were removed within 24 h of slaughter. Ethics permission for the collection of waste human semitendinosus tissue from patients undergoing patellar stabilisation surgery was obtained from Essex 2 Research Ethics Committee, reference 09/H0302/3. All samples were frozen at −20 °C until required.

Upon defrosting, sample hydration was maintained with Dulbecco’s modified Eagle’s medium (DMEM) while collagen fascicles of length greater than 20 mm were carefully dissected. A variation in the mechanical properties of fascicles from different regions of the human patellar tendon has previously been observed,^27^ and as a result, fascicles were only taken from the central regions of the bovine tendons. Previous studies have shown no difference in the mechanical properties of fascicles from any of the three bovine extensor tendons in the hoof so these were pooled.^28^ Fascicle diameters were measured continuously along a 10 mm length in the centre of each fascicle using a laser micrometer (LSM-501; Mitutoyo, Kawasaki, Japan; resolution of 0.5 µm). The minimum value of approximately 25 measurements over this 10 mm length was used to calculate the cross-sectional area assuming a circular cross section. Fascicles with a diameter between 0.18 and 0.4 mm were considered for mechanical characterisation.

### Stress relaxation and creep tests

Each fascicle was secured in an individual custom-made stainless steel loading chamber,^29^ with a grip-to-grip distance of 10 mm. The grip system ensured slippage of the fascicles did not occur during testing.^29^ Each chamber was filled with DMEM, to maintain full hydration during testing, and the chambers were secured within a BOSE loading frame (BOSE Corporation, Eden Prairie, MN, USA) housed in an incubator to maintain samples in 5% CO_2_ at 37 °C.

Legerlotz et al.^29^ have previously established that cyclic stress relaxation to 60% of the failure strain resulted in a significant decrease in fascicle mechanical properties in just 15 min. This value was subsequently adopted for testing in the current study, allowing a rapid analysis of damage accumulation across the range of tendon types; 60% strain to failure corresponded to 14% applied strain in all cases. During stress relaxation tests, cyclic loading at a frequency of 1 Hz with a sine wave form was carried out under displacement control, to a fixed peak displacement of 1.4 mm (initial gauge length of 10 mm). Cyclic loading was carried out for 5 min (300 cycles), 15 min (900 cycles) or 30 min (1800 cycles) and the reduction in load over time was monitored. Mean stress relaxation (defined as percentage of stress at cycle 1) was determined for the three time points, after which samples were subjected to confocal imaging or quasi-static tensile testing to determine the effects of fatigue loading.

Quasi-static tensile testing (Bionix100; MTS, Cirencester, UK; 50 N Load Cell) was carried out using the same DMEM filled stainless steel loading chambers. Testing was carried out at an extension rate of 1 mm/s to failure and stress–strain curves derived using the initial measured cross-sectional area. At least 10 fascicles from multiple tendons were tested at each analysis point: 0, 300, 900 and 1800 cycles of stress relaxation.

For creep tests, cyclic loading was again carried out at a frequency of 1 Hz (same sine wave form), but under load control to a constant load of 60% of the UTS. Based on previous quasi-static testing of fascicles from these three tendon types, an average UTS value of 48 MPa was adopted for the bovine extensor and flexor samples and 60.22 MPa for human semitendinosus samples, equating to an applied stress of 28.8 MPa in the case of the bovine fascicles and 36.1 MPa for the human tendon. Displacement was monitored through the duration of the test. At least 12 fascicles from four tendons were cyclically tested to failure for each of the three different tendon types. Additional tests were terminated after 300, 900 and 1200 cycles and samples were stored for confocal imaging.

### Confocal imaging

At each level of fatigue, fascicles were stained with acridine orange and imaged to investigate damage accumulation. Acridine orange is a nucleic acid selective fluorescent cationic dye used extensively for a variety of applications including the tracking of tenocyte nuclei during straining experiments under confocal microscopy.^6,30^ For nuclear staining, concentrations of the order of 5 µM are typically used; however, at higher concentrations, the staining becomes non-specific and will stain the collagen and matrix constituents. A 5 mM acridine orange (Invitrogen, Eugene, OR, USA) solution in DMEM was used to stain the fascicles for 40 min, followed by repeated phosphate-buffered saline (PBS) washing to ensure removal of excess stain. Imaging was carried out using a PerkinElmer Ultraview Spinning Disc Confocal system with a Nikon Eclipse TE300 Microscope and 4* and 20* objective apertures. Lower magnification images were used in order to construct a composite image of the entire fascicle length, while the higher magnification images enabled qualitative investigation of damage accumulation within the tendon matrix.

### Statistical analysis

Statistical significance in percentage stress relaxation as well as UTS values from quasi-static tests after cyclic loading was determined with a single-factor analysis of variance (ANOVA) followed by Tukey’s post hoc test (IBM SPSS software). For investigation of the number of cycles to failure during creep testing, a Kruskal–Wallis test followed by a Mann–Whitney test was used. For all statistical tests, significance was established at p≤ 0.05.

## Results

### Stress relaxation

**Figure 1.**
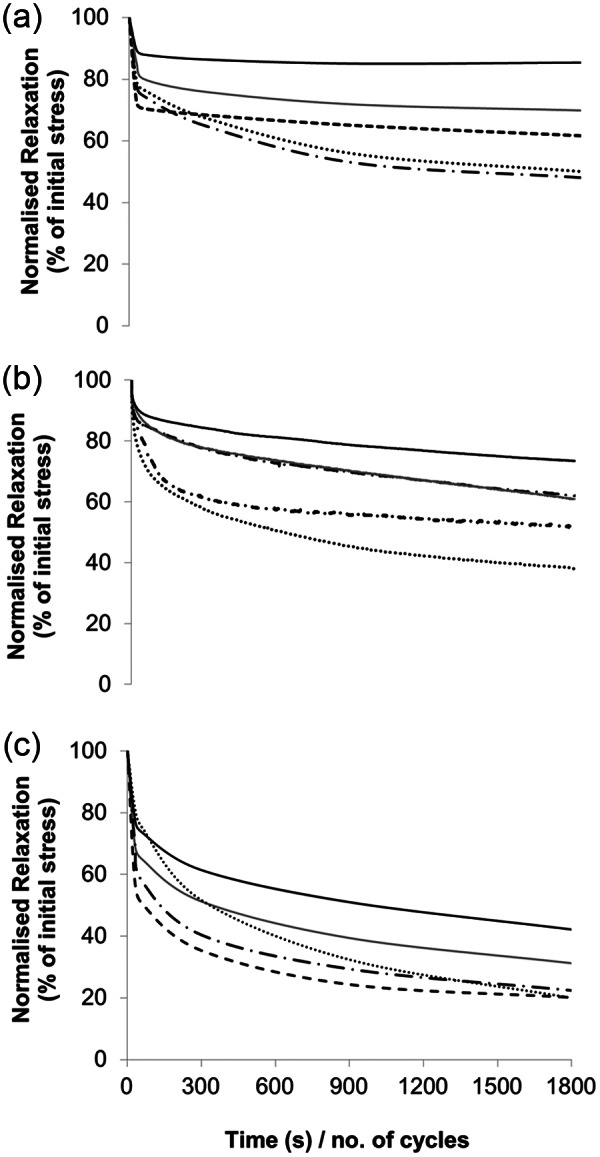
Typical stress relaxation curves from (a) human, (b) bovine flexor and (c) bovine extensor tendon fascicles.

Typical stress relaxation curves from each tendon type are shown in Figure 1 with mean data in Table 1. The data indicate markedly greater stress relaxation for the extensor tendon fascicles compared with the other two tendon types. After just 300 cycles, the sample stress had decreased by 48% in extensor fascicles compared to less than 30% in the human semitendinosus. The human semitendinosus and bovine extensor created the two extreme responses, with the energy-storing human semitendinosus exhibiting significantly more fatigue resistance throughout. However, a significant difference between the response of the two bovine tendons was only apparent at 1800 cycles and at no time point did a statistically significant variation exist between the human semitendinosus and bovine flexor tendon.

**Table 1.** Mean stress relaxation data ± standard deviation (expressed as a percentage of the stress applied in the first cycle) for human semitendinosus, bovine flexor and bovine extensor tendon after 300, 900 and 1800 cycles to a displacement at 60% UTS.

**Tendon**	**0 cycles**	**300 cycles**	**900 cycles**	**1800 cycles**
**Human Semitendinosus**	100%	70.89±11.82%	64.89±12.56%	61.50±13.95%
**Bovine Flexor Tendon**	100%	66.26±11.09%	57.99±15.87%	54.34±17.95%
**Bovine Extensor Tendon**	100%	52.12±14.91%	42.57±16.16%	36.06±17.20%

Statistical significance (p≤0.05) was observed between human semitendinosus and bovine extensor samples at all time points and between the bovine flexor and bovine extensor after 1800 cycles; difference between the human semitendinosus and bovine flexor remained insignificant at all time points.

**Figure 2.**
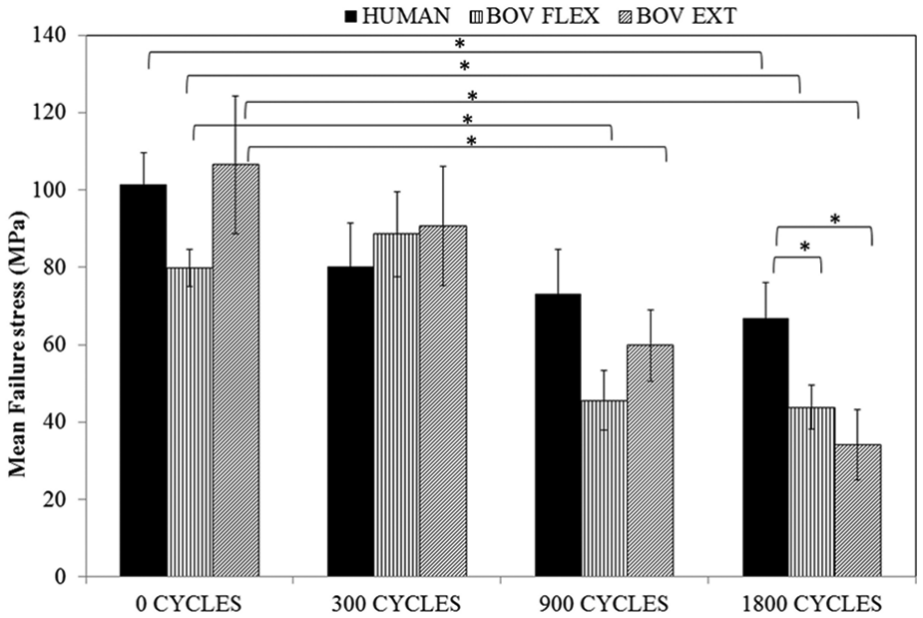
Mean failure stress for quasi-static tests to failure, carried out on samples after 300, 900 or 1800 cycles of stress relaxation. ‘*’ denotes significant variation at p≤0.05.

Despite considerable stress relaxation after 300 cycles of loading, all samples including the bovine extensor tendon fascicles retained their tensile strength during quasi-static testing at this first time point (Figure 2). However, by 900 cycles of stress relaxation, both bovine tendon types showed a statistically significant reduction in UTS, and by 1800 cycles, the decrease in UTS was also evident in human semitendinosus fascicles. Interestingly, although UTS tapered off most rapidly in the bovine extensor, it actually exhibited the highest mean UTS in control samples pre-fatigue testing. If UTS reductions with stress relaxation are expressed as a percentage of the starting value, the dramatic drop in strength in the bovine extensor becomes evident (66% at 1800 cycles) compared to the human semitendinosus (32% decrease).

**Figure 3.**
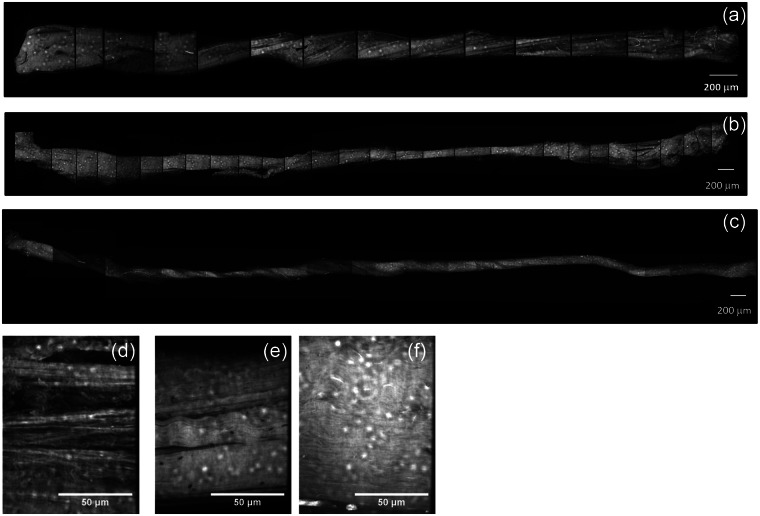
Confocal images of fascicles of (a and d) human, (b and e) bovine flexor and (c and f) bovine extensor tendons. All samples were stained with 5 mM acridine orange solution.

The confocal images of Figure 3 show fascicles from the three tendon types before loading. The low magnification composite images allow imaging of the entire fascicle length. Extensor fascicles could be extracted from the bovine tendon with ease and showed a generally consistent cross section along their length and less evidence of damage than the other two tendon types (Figure 3(c)). Bovine flexor fascicles appeared to show a much higher degree of interfascicular binding and were dissected with considerably more difficulty than the extensor fascicles (Figure 3(b)), while a degree of surgically induced damage appeared to be present in the semitendinosus fascicles (Figure 3(a)). At a higher magnification, all non-loaded fascicles generally exhibited a dense, well-aligned collagen fibrillar arrangement, although both human and flexor tendon fascicles exhibited some areas with widening of the inter-fibre space (Figure 3(d) and (e)).

**Figure 4.**
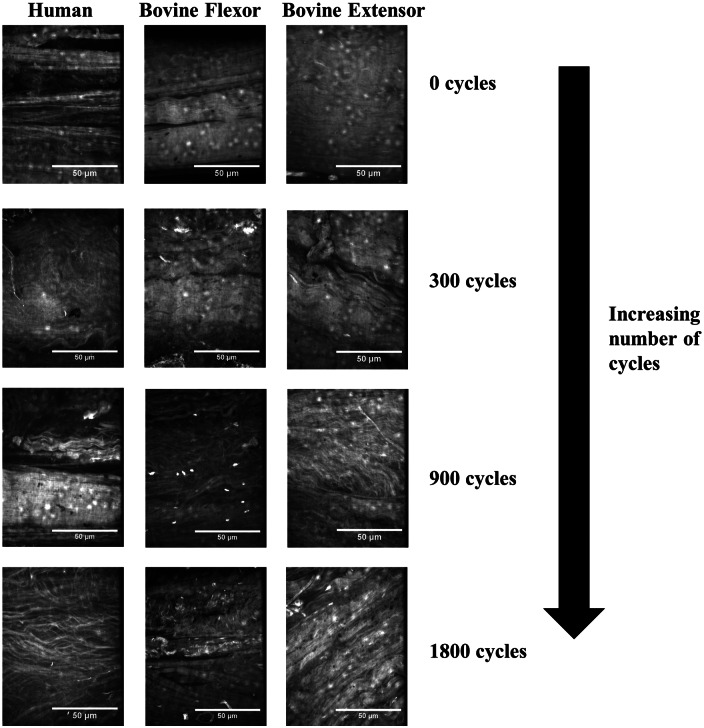
Collagen organisation in tendon fascicles with increasing number of cycles of stress relaxation.

Although all fascicle types retained their tensile strength after 300 cycles of stress relaxation, some degree of disorganisation within the collagen matrix structure was observed at this time point particularly with the bovine samples (Figure 4). After 900 cycles, significant kinking of the collagen fibrils was observed in all fascicle types, alongside some breakdown of collagen fibril alignment. While this correlated with the reduction in UTS in bovine samples, no significant decrease in the UTS of human tissue was evident at this time point. By 1800 cycles, the fascicles exhibit a more fibrous structure and spacing between fibres became evident; the clear evidence of matrix breakdown in all samples supports the significant loss in mechanical properties observed (Figure 4).

### Creep testing

**Figure 5.**
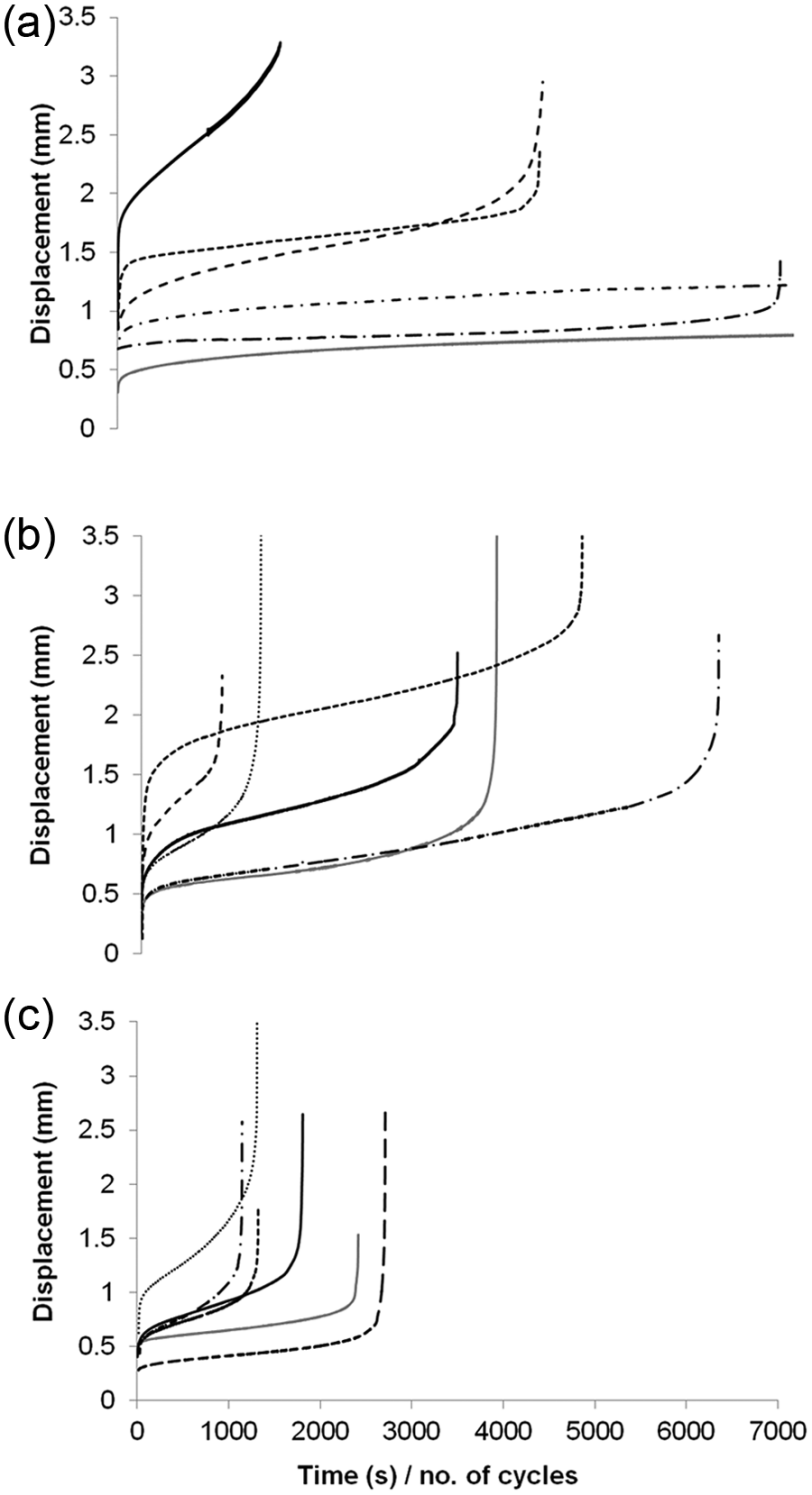
Typical creep curves for (a) human semitendinosus, (b) bovine flexor and (c) bovine extensor tendon.

**Figure 6.**
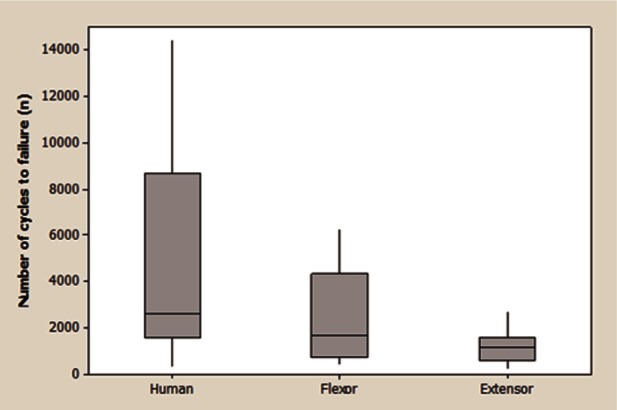
Box and whisker plot summarising number of cycles to failure during creep testing for the 3 tendon types. The horizontal line within the box indicates the median, boundaries of the box indicate the 25^th^ (Q1) and 75^th^ (Q3) percentile, the upper whisker indicates the highest data value within the limit Q3+1.5(Q3-Q1) and the lower whisker the lowest data value within Q1-1.5(Q3-Q1).

Typical creep curves for the three tendon types are shown in Figure 5, with mean cycles to failure presented in Figure 6. Large variations in the cycles to failure were evident for all three tendon types (Figures 5 and 6), but this was particularly true for the human semitendinosus where cycles to failure varied from 340 to in excess of 80,000. As a result of the large variances, significant difference in the cycles to failure existed only between the human and bovine extensor tendon samples. Creep curves for the three tendon types showed a similar form, although initial displacement and the length of stage II (secondary) creep varied within and between tendon types. No significant correlation was observed between initial displacement and number of cycles to failure for any of the tendon types. For bovine samples, the gradient of the secondary creep stage was relatively consistent across all samples; however, in human samples, an elevated gradient was observed in those samples failing at lower numbers of cycles.

**Figure 7.**
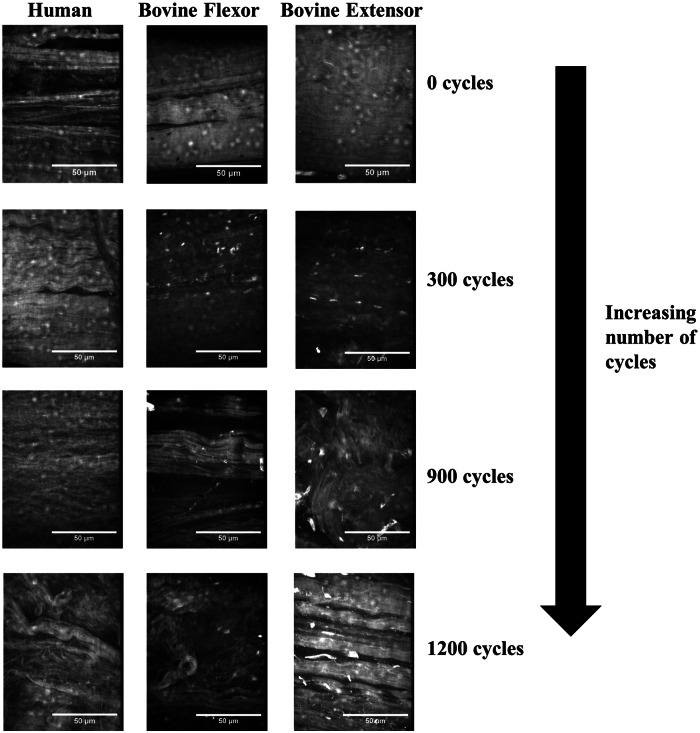
Collagen organisation in tendon fascicles with increasing number of cycles of creep loading.

While little accumulated damage was observed after 300 cycles in any of the three tendon types, very significant damage was observed in all tendons after 900 and 1200 cycles (Figure 7). Fibril kinking and loss of alignment were observed in all instances along with fibril breakages and widening of the inter-fibre space particularly after 1200 cycles. This damage generally appeared more significant than observed in stress-relaxed samples.

## Discussion

This is the first study to carry out a full comparison of fascicle level fatigue behaviour across both functionally distinct tendons and species, correlating mechanical changes with structural ones. It additionally provides the first data comparing stress relaxation and creep response in these different tissue types.

The bovine extensor tendon fascicles considered in the current study exhibited significantly less fatigue resistance under both cyclic stress relaxation and cyclic creep conditions than either of the other tendon types, but particularly when compared to the human semitendinosus. The bovine flexor tendon appeared to exhibit an intermediary response in both instances, although no significant variation was observed between semitendinosus and bovine flexor in terms of either stress relaxation or number of cycles to failure during creep.

Both the semitendinosus and bovine digital flexor tendons will have a degree of energy-storing capability, helping return energy to the muscle-tendon unit for more efficient movement. The extensor tendon on the other hand acts only to extend the distal limb prior to limb placement. A clear difference in the mechanics of digital flexor and extensor tendons has previously been observed in the horse,^8,31^ but this is in an animal where strains of 16% have been observed in the superior digital flexor tendon during galloping compared to a maximum peak strain of just 2.5% in the extensor tendon.^32^ The variation in strains (and associated loads) between flexor and extensor tendons is likely to be less significant in the cow where sustained high-speed movement is less common, and a less significant adaptation could perhaps be expected in the two tendon types. This supports the absence of significant variation in cycles to failure and stress relaxation at all but the last time point between bovine flexor and extensor fascicles. Much like the equine flexor, the human semitendinosus is expected to be well adapted to cope with the relatively high strains and loads it experiences, hence the superior fatigue characteristics.

An area of concern in the consideration of fascicle mechanics is the effect of fascicle dissection and the influence of any resultant damage on mechanical characteristics. The fascicles of the bovine digital extensor tendon are visible to the naked eye and run in a straight line along the length of the tendon, making for straightforward dissection.^16^ Flexor tendon fascicles, on the other hand, are much less visible, are not always straight along the length of the tendon and also appear to exhibit much more interfascicular binding. These factors can result in the generation of quite significant dissection damage of flexor tendon fascicles and may explain the wider spread of cycles to failure observed with flexor fascicles compared to extensor. It appears that while the flexor tendon exhibits fatigue behaviour most representative of the human semitendinosus, it is also far more likely to suffer from significant dissection damage. Having said this, the range of cycles to failure was even more significant for the human semitendinosus fascicles, which did not appear to exhibit the same difficulty in dissection. However, these samples may be influenced by the necessary surgical removal.

As a starting point for this study and based upon previous quasi-static testing of fascicles, an average UTS value of 48 MPa was assumed for the bovine tendon fascicles and 60.22 MPa for the fascicles from the human semitendinosus, with 60% of these values used for creep testing. However, when quasi-static testing was carried out on freshly dissected fascicles for the current study, UTS values for both the bovine extensor and human semitendinosus were found to be in excess of 100 MPa and the bovine flexor greater than 75 MPa. Actual UTS values for the three tendon types before loading are included in Table 2, which also includes the percentage of this UTS used during creep testing. These data highlight the potential variation in both quasi-static and fatigue properties that can be induced through sample preparation and may explain the high sample variability.

**Table 2.** Mean UTS Values ± standard deviation, determined during the course of this work for tendon fascicles without fatigue loading and actual percentage of the UTS applied during creep testing.

**Tendon type**	**UTS (MPa)**	**Percentage UTS applied during creep**
**Human Semitendinosus**	101.27±26.29	35.6%
**Bovine Flexor**	78.78±22.24	36.6%
**Bovine Extensor**	107.77±55.05	26.7%

UTS: ultimate tensile strength.

Standard deviations are included in parenthesis.

The elevated quasi-static tensile strength of the bovine extensor is at first glance surprising given the low in-life loading of this tendon type. While this may to a degree be the result of the absence of dissection damage in these fascicles, previous studies have also found a lack of correlation between UTS and in-life loading.^20,26,33^ In the equine model, at both the whole tendon and fascicle level, a significantly lower UTS has been observed in the SDFT than the CDET.^26^ It is suggested that in the energy-storing tendon, some compromise in UTS is observed in order to allow for the reduced modulus and higher extensibility necessary for energy storage and transfer.^26^

It should be noted that despite human and bovine flexor tendon fascicles showing a greater degree of initial damage, they still showed superior fatigue characteristics; differences may well be more significant in the absence of damage. Furthermore, the use of a lower proportion of the UTS in the case of the bovine extensor tendon only highlights its reduced fatigue resistance compared to other two tendon types. It has been shown previously that when loading is carried out at levels proportional to in-life loading, then widely varying tendon types generally exhibit a similar number of cycles to failure.^34^ The human semitendinosus is likely to see a stress higher than that utilised here and the bovine extensor considerably less; hence, balancing these for in-life load would be expected to result in much more consistent cycles to failure.

The confocal images, although undoubtedly qualitative, provide a good insight into the damage accumulation that occurred during both creep and stress relaxation tests. In previous studies, the accumulation of matrix damage has been imaged using a second harmonic image technique^35,36^ and images obtained in this manner are not dissimilar to those obtained here. A useful description of the accumulation of damage in response to creep loading during a controlled in vivo study was included in the article of Neviaser et al.^36^ and is summarised in Table 3.

**Table 3.** Progression of fatigue damage during controlled in vivo loading: the effect on collagen fibril architecture.

Level of fatigue	Collagen fibril architecture
No fatigue	Aligned collagen fibrils
Low-level fatigue	Kinked fibre deformations
Moderate fatigue	Kinked fibre deformation with widening of the inter-fibre space
High-level fatigue	Severe matrix disruption, fibre thinning, angulations and fibre discontinuities

Source: Data collated from Neviaser et al.^36^

These stages provide not only a very good description of the progression of damage observed here in Figures 4 and 7 for creep but also stress relaxation loading. The initial stages of kinked fibres and widening of the inter-fibre space suggest the inter-fibre space forms the weakest link of the tendon fascicle matrix and fatigue damage initiates here. Indeed, after 300 cycles of stress relaxation, the only damage was generally in the form of kinked fibres, and quasi-static testing would suggest this did not affect the tensile characteristics of the fascicles from any of the three tendon types. Only with high levels of fatigue was evidence of breakdown of the collagen composite structure observed. In the human tendon fascicles, it was not until 1800 cycles of stress relaxation, with quite significant matrix damage, that a statistically significant reduction in UTS was observed. Despite the more prolonged maintenance of mechanical strength in the energy-storing tendons, the nature and rate of matrix breakdown with cyclic deformation did not differ significantly between the three tendon types.

Although the appearance of the fatigue damage was similar for the two testing techniques, creep tested samples demonstrated more significant damage at a lower number of cycles. This was observed across the three tendon types. It has been speculated that creep is fundamentally more non-linear than relaxation, and that the microstructural processes taking place during the two testing routes could be fundamentally different.^37^ During creep, fibre bundles are recruited and stretched out progressively. Clearly, this progressive recruitment would not be expected to occur under stress relaxation, where extension is not increased during the course of the test, perhaps explaining the more significant, widespread matrix damage observed in creep. Nevertheless, with both testing conditions, damage appeared to be initiated in the non-collagenous matrix.

The matrix changes appear broadly consistent with the fairly limited data from tendinopathic biopsies.^38–41^ Tendinopathic and ruptured tendons have been observed to exhibit fibres with increased spacing and waviness, as well as disrupted crimp continuity.^14^ Not only are the microstructural changes in tissue biopsies generally consistent with those in fatigue models, but matrix changes have also been observed to precede cellular, molecular and mechanical changes.^14^ Kongsgaard et al.^41^ observed that while fibril morphology was abnormal in tendinopathic biopsy specimens, in vivo tendon mechanical properties did not appear altered.

The assessment of fatigue behaviour in the current study highlighted the care needed in dissection. However, even with appropriate dissection technique, fatigue characteristics should not be assumed to be defined at the fascicular level. In a study of the equine SDFT and CDET that considered both the fascicular and whole tendon level, significantly greater extensions were observed in whole flexor tendons than their constitute fascicles, behaviour facilitated by greater capacity for sliding at the interfascicular interface.^26^ The same degree of sliding was not observed in the extensor fascicles. In several studies comparing the behaviour of whole tendon and fascicles, higher failure stress and modulus have been observed in the whole tendon compared to the fascicle,^26,42^ contrary to standard composite models that would predict a lower stiffness for the bulk material. While interfascicular behaviour may in part explain differences between whole tendon and fascicle behaviour, fascicle isolation is also likely to have an effect. There are, however, distinct advantages to fascicle testing: besides providing an understanding of mechanics at levels below the whole tendon, it also allows for far more straightforward imaging, not only of matrix damage as demonstrated here but also the effect of fatigue on cellular morphology and processes. Fascicular testing also allows for multiple testing conditions to be considered with fascicles from the same animal (intra-animal comparison) and has thus been used in a number of studies of mechanobiology and load-induced matrix metabolism.^29,43,44^

This study provides quantitative data concerning stress relaxation and creep response and a qualitative assessment of the accumulation of damage at the larger hierarchical levels of the tendon structure (primarily at the fibre level). It is deformation and structural changes at this level that are perhaps the most significant for the cell environment. The kinking of the collagen fibres and widening of the inter-fibre space in the earlier stages of fatigue are likely to elicit a response from the resident cells, which may well take an active role in controlling further tendon behaviour and may trigger reactive tendinopathy in vivo.^45^ An understanding of the interactions between collagen fibres, cells and the inter-fibre matrix and how these are influenced by fatigue damage is therefore of critical importance. In an evaluation of human asymptomatic tendons, it has been found that cell change is always observed alongside matrix change. In addition, the matrix change was found to be largely in the inter-fibre space and only in more extreme damage was a change in the collagen structure observed.^46^ Fatigue damage will have a significant effect on the cell environment and biological activity, and fatigue testing of viable samples is thus a critical next step.

## Conclusion

The bovine extensor tendon, with its positional role, demonstrated a poorer fatigue response than the more highly loaded bovine flexor or the human semitendinosus tendons, supporting previous data, suggesting that fatigue resistance correlates with in-life loading. Despite the superior fatigue response of the flexor and semitendinosus tendon fascicles, a very similar degree of matrix breakdown was observed in the confocal images of all three tendon types. Indeed, some damage to the matrix structure could be observed, without detriment to the quasi-static properties of the fascicle.
